# Phospholipid-Based Prodrugs for Colon-Targeted Drug Delivery: Experimental Study and In-Silico Simulations

**DOI:** 10.3390/pharmaceutics11040186

**Published:** 2019-04-16

**Authors:** Milica Markovic, Arik Dahan, Shahar Keinan, Igor Kurnikov, Aaron Aponick, Ellen M. Zimmermann, Shimon Ben-Shabat

**Affiliations:** 1 Department of Clinical Pharmacology, School of Pharmacy, Faculty of Health Sciences, Ben-Gurion University of the Negev, Beer-Sheva 8410501, Israel; milica@post.bgu.ac.il (M.M.); arikd@bgu.ac.il (A.D.); 2 Cloud Pharmaceuticals Inc., Durham, NC 27709, USA; skeinan@cloudpharmaceuticals.com (S.K.); igor@cloudpharmaceuticals.com (I.K.); 3 Department of Chemistry, University of Florida, Gainesville, FL 32603, USA; aaron.aponick@gmail.com; 4 Department of Medicine, Division of Gastroenterology, University of Florida, Gainesville, FL 32603, USA; Ellen.Zimmermann@medicine.ufl.edu

**Keywords:** colon-targeted drug delivery, prodrug approach, ulcerative colitis, Phospholipase A_2_, molecular dynamics

## Abstract

In ulcerative colitis (UC), the inflammation is localized in the colon, and one of the successful strategies for colon-targeting drug delivery is the prodrug approach. In this work, we present a novel phospholipid (PL)-based prodrug approach, as a tool for colonic drug targeting in UC. We aim to use the phospholipase A_2_ (PLA_2_), an enzyme that is overexpressed in the inflamed colonic tissues of UC patients, as the PL-prodrug activating enzyme, to accomplish the liberation of the parent drug from the prodrug complex at the specific diseased tissue(s). Different linker lengths between the PL and the drug moiety can dictate the rate of activation by PLA_2_, and subsequently determine the amount of free drugs at the site of action. The feasibility of this approach was studied with newly synthesized PL-Fmoc (fluorenylmethyloxycarbonyl) conjugates, using Fmoc as a model compound for testing our hypothesis. In vitro incubation with bee venom PLA_2_ demonstrated that a 7-carbon linker between the PL and Fmoc has higher activation rate than a 5-carbon linker. 4-fold higher colonic expression of PLA_2_ was demonstrated in colonic mucosa of colitis-induced rats when compared to healthy animals, validating our hypothesis of a colitis-targeting prodrug approach. Next, a novel molecular dynamics (MD) simulation was developed for PL-based prodrugs containing clinically relevant drugs. PL-methotrexate conjugate with 6-carbon linker showed the highest extent of PLA_2_-mediated activation, whereas shorter linkers were activated to a lower extent. In conclusion, this work demonstrates that for carefully designed PL-drug conjugates, PLA_2_ overexpression in inflamed colonic tissues can be used as prodrug-activating enzyme and drug targeting strategy, including insights into the activation mechanisms in a PLA_2_ binding site.

## 1. Introduction

Prodrugs are derivatives of active drug moieties with little or no pharmacological effect, which undergo enzymatic and/or chemical conversion in the body into the active parent drug in order to accomplish an anticipated pharmacological effect [1,2]. The prodrug approach is often employed in order to overcome poor physicochemical properties and improve biopharmaceutical performance (i.e., absorption, distribution, metabolism, and excretion (ADME) properties) [3]. Additionally, prodrugs can be designed to overcome formulation obstacles and promote simpler administration, improve the effectiveness of the drug, enable site-specific drug delivery, and reduce toxicity [4,5]. Prodrugs can be hydrophilic or lipophilic, namely the drug lipophilicity is lower or higher than that of the parent drug [6]. Lipidic prodrugs consist of the drug moiety covalently bound to the lipid moiety, such as a fatty acid, triglyceride, steroid, or phospholipid (PL) [7,8]. An advantage of lipidic prodrugs is that a carefully designed prodrug can be incorporated into the physiological metabolic pathways for lipids and cross the barriers in the body, which are difficult to overcome otherwise.

Inflammatory bowel disease (IBD) is an umbrella term for a group of inflammatory disorders of the bowel, consisting mainly of Crohn’s disease (CD) and ulcerative colitis (UC) [9]. The main characteristic of UC is that the inflammation is localized in the mucosa, and it is generally limited to the colon [10,11]. Since the localization of the inflammation in UC patients is mainly in the colon, orally administered drugs have the potential to target the active drug moieties directly at the site of their action. However, drug targeting solely to the inflamed tissues, while preventing systemic side effects, remains a major challenge. Currently, the most common drug delivery approach in UC is colon drug targeting. Numerous formulation strategies have been developed to accomplish colon targeting, relying on rectal products or pH-, microflora-, time-, or pressure-dependent drug delivery. Rectal products are not always an adequate treatment option, since they don’t affect the entire colon, but rather only lower parts of the distal colon and rectum, as well as presenting the issues of difficult administration and poor patient compliance. Oral drug administration is more convenient route of administration, and is based on colon-targeting formulations or chemical modifications (such as the prodrug approach) [12]. The colonic microenvironment has few main characteristics that can be exploited in the design of the colon-targeted formulation approaches. The colonic microflora, pH, and pressure differences play an important role in the release of drugs from their biodegradable coating [13,14]. However, formulation development is based on the colonic conditions of a healthy colon, whereas each parameter (colonic microflora, pH, transit time, pressure) can be significantly altered in the diseased colon of IBD patients, and drug release can be largely affected by these changes [15]. A successful strategy for drug delivery in UC has been the prodrug approach [16,17]. It includes prodrugs of 5-aminosalicilic acid (5-ASA), bound to an additional moiety through an azo-bond, where bacterial azoreductases located in the colon can activate the 5-ASA prodrug and selectively liberate free 5-ASA in the colon [18,19]. These pharmaceutical strategies target the entire colon, regardless of where the inflammation in the colon is localized, but in many cases the inflammation occurs outside of the particular targeted region. In CD, the inflammation is present in the form of patches and can spread throughout the entire gastrointestinal tract (GIT). Colon targeting on its own can fail to accomplish the optimal pharmacological effect. Drug targeting to the inflamed tissues only, at the same time avoiding non-inflamed intestinal segments, is an important innovation in the treatment of IBD.

**Figure 1:**
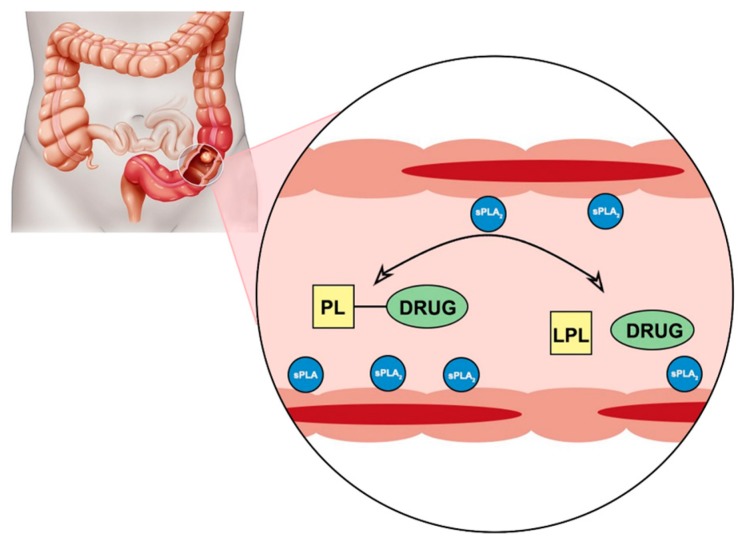
Illustration of colon-targeted drug delivery with phospholipid (PL)-drug conjugates; prodrug is activated by the enzyme sPLA_2_, overexpressed in the inflamed intestinal tissues of IBD patients. sPLA_2_, secreted phospholipase A_2_; IBD, inflammatory bowel disease.

Phospholipase A_2_ (PLA_2_) is an enzyme that is known to be overexpressed in the tissues of different inflammatory diseases [20,21,22,23,24] and in cancer [25,26,27,28,29]. It has been previously demonstrated that there is a significant overexpression of the PLA_2_ enzyme (including gene expression and protein content resulting in increased enzymatic activity) in the inflamed tissues of UC patients [20,21,22,23,24]. In addition, evidence of correlation between PLA_2_ biosynthesis and the degree of the bowel inflammation was also reported [21]. We, and others, have been advocating the use of a PL-prodrug approach to target PLA_2_ in inflamed tissues [30,31,32,33,34,35,36]. PLA_2_ hydrolyzes the fatty acid in the *sn*-2 position of the PL, thus releasing a free fatty acid and *sn*-1 lysophospholipid. It does not have selectivity towards a particular fatty acid, which provides a window of opportunity for the smart design of a PL-drug conjugate, which could provide activation by PLA_2_ enzyme, and result in liberation of the parent drug from the prodrug complex at the specific site of overexpression (Figure 1) [37]. We previously proposed a PL-prodrug design that consists of PL and *sn*-2 positioned drug moiety connected through a carbonic linker [32,38,39,40,41]. It was established that the linker length plays a crucial role in the extent of PLA_2_-mediated activation and subsequently determines the amount of drugs at the site of action [32,38,39,40]. The *sn*-2 position of the PL is quite resistant to spontaneous hydrolysis, therefore, PLA_2_-mediated prodrug activation can provide controlled and/or targeted delivery of the parent drug at the site of PLA_2_ overexpression. 

The focus of this manuscript is the prospective of the PL-prodrug approach and its utilization for colon-specific targeting in colitis. The feasibility of the presented PL-prodrug approach is studied with PL-fluorenylmethyloxycarbonyl (Fmoc) conjugates in vitro. Consequently, in silico calculations of the PL-methotrexate conjugate were done in order to determine the optimal linker length for this clinically relevant drug in UC. We designed, synthesized, and characterized prodrugs consisting of the PL and Fmoc moieties, with carbamate linkers between them. Two different linker lengths between the PL and the Fmoc were evaluated, and PLA_2_-mediated activation of the PL-Fmoc conjugates was studied in vitro. Additionally, we used a previously developed molecular modeling simulation technique to estimate the relative activity of PLA_2_ enzymes toward PL-prodrug molecules with different linker lengths [38,40]. The computational approach is based on thermodynamic integration and a molecular dynamics simulation of the transition state of the conjugate in the PLA_2_ enzyme complex. The model drug for these calculations was the immune system suppressant methotrexate. Simulations were done with both bee venom and human PLA_2_ enzymes to confirm the biorelevance of extrapolation from bee venom to human enzymes, as previously published [38,40]. The calculations showed that the free energy of the PL-prodrug binding to the transition state geometry of the enzyme determines the rate of PLA_2_-mediated activation. Our method allows optimization of the chemical structure of the molecular linker connecting drug moiety to the PL and could reduce the amount of chemical synthesis needed for the development of effective prodrugs. 

Newly synthesized conjugates of PL-Fmoc can provide the principle validation of the PL-based prodrug approach, and verification that this approach has the practical potential to be effective in inflamed tissues overexpressing PLA_2_ (e.g., colonic tissues in UC). In silico modeling studies were then conducted on methotrexate, as one of the drugs for the treatment of UC, these highly reliable computational methods may lead to the development of PL-based prodrugs containing the drug moieties already in use for UC. This work may become the foundation for a novel colonic drug-targeting option in the treatment of UC.

## 2. Materials and Methods

### 2.1. Materials

All chemicals were obtained from commercial suppliers and were used without further purification unless noted otherwise. Dichloromethane and 4-dimethylaminopyridine (DMAP) were distilled from calcium hydride. Lysophosphatidylcholine (LPC) was purchased from Nattermann Phospholipid GmbH, Cologne, Germany. Linkers-Fmoc: aminohexanoic acid and aminocaprylic acid were purchased from Sigma-Aldrich, Rehovot, Israel. Dextrane sulphate sodium was purchased from TDB Consultancy AB, Uppsala, Sweden. Anti-sPLA_2_ antibody was purchased from Abcam (Cambridge, MA, USA), and anti-GAPDH antibody from Santa Cruz Biotechnology (Dallas, TX, USA). Secondary donkey anti-rabbit IgG antibody was purchased from Santa Cruz Biotechnology (Dallas, TX, USA), and ECL reagent from Biorad, Rishon LeTsiyon, Israel.

### 2.2. Synthesis of Lysophosphatidylcholine-Amine-Fmoc Conjugates

**Scheme 1:**
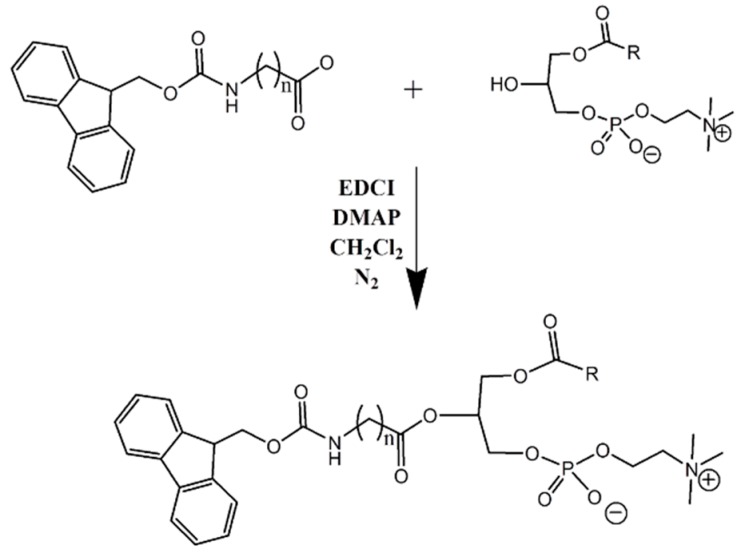
Prodrugs synthesis: Lysophosphatidylcholine (LPC)-aminohexanoic acid-fluorenylmethyloxycarbonyl (Fmoc) (compound 1, *n* = 5), and LPC-aminocaprylic acid-Fmoc (compound 2, *n* = 7).

In this study, two phosphatidylcholine-Fmoc conjugates were designed and synthesized (Scheme 1). Reactions involving air or moisture sensitive reagents were carried out under nitrogen. Column chromatography was performed using Merck silica gel 60 (70–230 mesh). Nuclear magnetic resonance (1H NMR) spectra were recorded on a Bruker DMX-500 operating at 500.1 MHz. Mass spectra were obtained with ESI-MS, API 2000 instrument (MDX, SCIEX, Concord, ON, Canada).

#### 2.2.1. General Synthesis Procedure

Condensation of LPC and linker-Fmoc: LPC (1 eq) dissolved in 30 mL dry dichloromethane, was mixed with linker-Fmoc (1 eq), EDCI (3 eq), and DMAP (2 eq) and stirred under nitrogen for 2 h, at room temperature (RT). Work up was carried out with 20 mL HCl 1 N. The reaction mixture was evaporated under reduced pressure. For purification, column chromatography was performed using Merck silica gel 60; Mobile phase: chloroform:methanol:acetic acid (95:5:0.5 *v*/*v*). TLC solvent system: chloroform:methanol:acetic acid (60:40:0.1 *v*/*v*).

#### 2.2.2. Data Specification for LPC-Linker-Fmoc Complexes

Compound **1** (LPC-aminohexanoic acid-Fmoc) was prepared from 187 mg LPC (0.37 mmol), 130 mg aminohexanoic acid-Fmoc (0.37 mmol), 212 mg EDCI (0.111 mmol) and 90 mg DMAP (0.74 mmol) in 30 mL dry dichloromethan (CH_2_Cl_2_). Workup: evaporation of the solvent, washing, and separation with 1 N HCl and chloroform, and finally evaporation of the organic phase by rotary evaporator. Column chromatography was performed to give a yield of 215 mg (317%). 1H-NMR (500 MHz; CDCl3); δppm; 0.810 (s, 3H); 1.184 (m, 24H); 1.837 (s, 4H); 2.259 (s, 4H); 3.131 (s, 2H); 3.578 (m, 11H); 7.19 7(m, 12H); 8.110 (s, 4H). LC-ESI-MS (ESI): *m*/*z* = 830.49 (M−H).

Compound **2** (LPC-aminocaprylic acid-Fmoc) was prepared from 200 mg LPC (0.39 mmol), 149 mg aminocaprylic acid-Fmoc (0.39 mmol), 224 mg EDCI (1.17 mmol), and 95 mg DMAP (0.78 mmol) in 30 mL dry dichloromethan (CH_2_Cl_2_). Workup: evaporation of the solvent, washing, and separation with 1 N HCl and chloroform, and finally evaporation of the organic phase by rotary evaporator. Column chromatography was performed to give a yield of 180 mg (51.6%). 1H-NMR (500 MHz; CDCl3); δppm; 0.810 (s, 3H); 1.215 (m, 36H); 3.13 (s, 13H); 6.622 (s, 2H); 7.197 (s, 4H); 8.110 (s, 2H). LC-ESI-MS (ESI): *m*/*z* = 859.52 (M−H).

### 2.3. Enzymatic Activity

Hydrolysis assay of the LPC-amine-Fmoc complexes using bee venom PLA_2_ followed a previously described protocol [32]. Briefly, PL-Fmoc complexes (0.09 mg) were dissolved in DMSO (50 μL), in different tubes, and were then added to 950 μL of Tris buffer, containing Tris-HCl 25 mM, CaCl_2_ 10 mM and NaCl 300 mM (pH 7.4) containing 1.24 units/mL PLA_2_ obtained from bee venom. This mixture was incubated for 105 min at 25 °C. Samples were collected in intervals after 0, 5, 25, 45, 65, 85, and 105 min. The samples were developed in parallel with the reference compound (phosphatidylcholine), and the reaction was terminated by addition of methanol in 1:1 *v*/*v* and was immediately frozen.

### 2.4. sPLA_2_ Expression in Dextrane Sulphate Sodium Model of Colitis

A dextrane sulphate sodium (DSS) model of acute colitis in rats was developed using 5% DSS (molecular weight, 40,000 kDa) administered in their drinking water for 10 days, according to previously published protocols (*n* = 2) [42,43]. The control animals drank sterilized water only. Clinical progression of colitis was evaluated using the disease activity index (DAI) score, which includes the following parameters: weight loss compared to initial weight, stool consistency, and rectal bleeding. DAI parameters are defined as: weight loss 0 (no loss), 1 (1–5%), 2 (5–10%), 3 (10–20%), and 4 (>20%); stool consistency: 0 (normal), 2 (loose stool), and 4 (diarrhea); and bleeding: 0 (no blood), 1 (hemoccult positive), 2 (hemoccult positive and visual pellet bleeding), and 4 (gross bleeding, blood around anus) [42]. On day 10, the rats were anesthetized by intramuscular injection of 1 mL/kg of ketamine-xylazine solution (9%:1%). The abdomen was opened by a midline incision of 3–4 cm, followed by the colon removal. Colonic mucosa was scraped on ice-cold glass. Immunoblot analysis of the mucosal samples was performed by a previously described method [44]. Colonic tissues were suspended in lysis buffer (150 mM sodium chloride, 50 nM TrisHCl pH 7.4, 1 mM EDTA, 1% Triton X-100, 1% sodium deoxycholic acid, 0.1% SDS). Protease (1:50) and phosphatase inhibitors (1:100), (Sigma-Aldrich Israel, Rehovot, Israel) were added just before using the lysis buffer. Homogenization (Polytron) was done for 30 s, 4 times, following ultrasonication for 10 s on ice. The homogenate was then centrifuged at 12,000× *g* for 30 min at 4 °C, and the supernatant was used for protein content determination (Bradford assay) and immunoblotting. Samples were resolved in 15% sodium dodecylsulphates gel electrophoresis, followed by electrophoretic transfer onto a Hybond ECL nitrocellulose membrane. The membrane was blocked for 1 h in TTBS solution with 3% milk, followed by overnight incubation at 4 °C with rabbit polyclonal anti-PLA_2_ antibody (1:1000). The secondary antibody was donkey anti-rabbit IgG (1:5000). Equal protein loading was ensured by normalization to GAPDH. Detection of immunoreactive bands was carried out using enhanced chemiluminescence (Biological Industries, Beit-Haemek, Israel). Semi-quantitative analysis was carried out using a computerized image analysis system (Image Studio Lite Ver. 5.2, LI-COR, Lincoln, NE, USA).

### 2.5. Analytical Methods

A high-performance liquid chromatography (HPLC) system Waters 2695 Separation Module, (Waters Inc., Milford, MA, USA). with a Waters UV 2996 Photodiode Array UV Detector, (Waters Inc., Milford, MA, USA) was used for determining in vitro activation of the different conjugates by PLA_2_. Identification of the complex was carried out with a RP-18 column and confirmed by UV and co-injection with an authentic sample. The HPLC conditions were as follows: Thermo SCIENTIFIC Part No. 25005-254630 Hypersil gold Dim column 250 × 4.6, particle Size 5 µm, SN: 08830609, LOT: 9278 (Thermo Fisher Scientific, Waltham, MA, USA), an isocratic mobile phase containing isopropanol:water:methanol (70/22/8 *v*/*v*), and acetic acid 0.1% in 20 min at the flow rate of 1 mL/min, and the detection wavelength was 265 nm.

### 2.6. Mass Spectrometry

The LC-ESI-MS system was API 2000 Instrument (MDX, SCIEX, Concord, ON, Canada). The sample (1 μg/mL) was injected directly to the ESI ion source (negative mode) in a rate flow of 10 μL/min (the solvent system was 50:50 methanol:water).

### 2.7. Statistical Analysis

In vitro experiments were *n* = 3, and the values are expressed as average ± standard deviation (SD). Statistically significant differences among experimental groups were determined using a nonparametric Kruskal-Wallis test for multiple comparisons, and a two-tailed non-parametric Mann—Whitney *U* test for two-group comparison; *p* < 0.05 was termed significant.

### 2.8. Computations of Relative Activity of PL-Drug Variants in PLA_2_

We have previously published the computational methods in detail [38,40]. The aim of this study is to use the PLA_2_ enzyme in order to hydrolyze the *sn*-2 acyl bond of the PL-prodrug molecule and release a free drug to the colon. For the PLA_2_ cleavage reaction to occur, a prodrug molecule should adopt the well-defined transition state geometry within the enzyme, which is characterized by strong interactions of the *sn*-2 carbonyl oxygen with the calcium atom (in the PLA_2_ active site), as well as the specific position of the protein His residue, which activates a water molecule for the nucleophilic attack on the prodrug acyl bond. Briefly, concentration of the prodrug in the transition state geometry with PLA_2_ and the rate of the cleavage reaction are determined by the free energy of the binding of the prodrug in the enzyme active site.

(1) $$K_{hydr}\sim C_{tr\;geom}\sim exp\left(-\Delta G^{tr}/RT\right)$$

**Figure 2:**
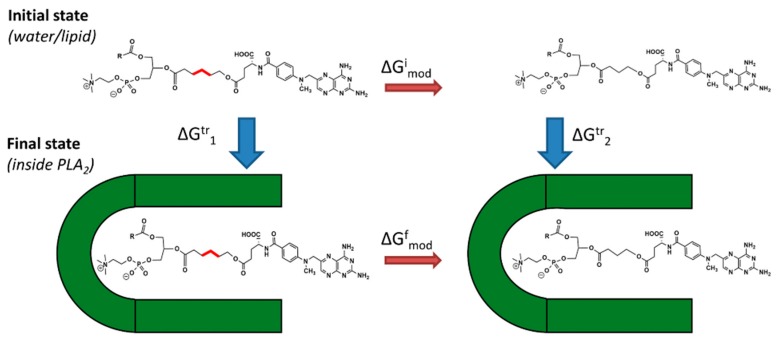
Diagram of the thermodynamic cycle used to compute the relative binding energies of PL-methotrexate molecules in the transition state complex of PLA_2_. $\Delta G_{mod}^{f}$ and $\Delta G_{mod}^{i}$ are free energies of adding or removing of -CH_2_ units in the initial and final states; $\Delta G^{tr}{}_{1}$ and $\Delta G^{tr}{}_{2}$ are the binding free energies of the prodrug molecules in the transition state geometry of PLA_2_.

Equation (1) represents proportional values of $K_{hydr},$ the rate of the hydrolysis of prodrug sn-2 acyl bond; $C_{tr\;geom}$, the concentration of the prodrug and $\Delta G^{tr}$, and the binding free energy of the prodrug in the PLA_2_ transition state geometry. PLA_2_-mediated hydrolysis rates are evaluated by changing the prodrug linker lengths. Relative binding energies were calculated by computing the free energies’ changes that occur upon the linker length change in two states (initial state-free prodrug molecules in solvent or lipid phase; final state prodrug-PLA_2_ in the transition state geometry). Linker length is changed using alchemical transformation and the changes in free energy are calculated using a thermodynamic cycle (Figure 2). The computed value is $\Delta \Delta G^{tr}$ and it presents the changes in PLA_2_ transition state binding free energy of the PL-prodrug associated with decreasing/increasing linker length. $\Delta \Delta G^{tr}$ values were calculated (Equation (2)), from differences in the free energy in the final state $\Delta G_{mod}^{f}$ (inside PLA_2_) and in the initial state $\Delta G_{mod}^{i}$ (in water or lipid phase):

(2) $$\Delta \Delta G^{tr}=\Delta G_{2}^{tr}-\Delta G_{1}^{tr}=\Delta G_{mod}^{f}-\Delta G_{mod}^{i}$$

$\Delta G_{mod}^{f}$ and $\Delta G_{mod}^{i}$, free energies of adding or removing of hydrocarbon linker units in the initial and final states, were computed in two steps. During the first step, the free energy of removing the linker CH_2_ units were computed using the thermodynamical integration (TI) method and keeping the distance between the end of the drug moiety and the new linker end constrained. In the second step, free energy associated with attaching the drug moiety to the shortened linker was computed using the umbrella sampling (US) and weighted histogram analysis method (WHAM) methods by progressively applying different harmonic constraints between linker end and the drug moiety. Thermodynamic integration (TI) computes the difference in free energy between two given states by ensemble-averaging enthalpy changes along the path connecting two states. The umbrella sampling (US) method involves applying a set of harmonic constraints between atoms in the system and observing changes in the average distances between constrained atoms. The series of umbrella sampling simulations were analyzed using the weighted histogram analysis method (WHAM).

### 2.9. Preparation of Molecular Models 

PL-methotrexate prodrugs with linker lengths varying from 2 to 8 carbons were considered. Two PLA_2_ variants were also studied: bee venom PLA_2_—The enzyme which was used in the in vitro experiments of PLA_2_-mediated activation of PL prodrugs, and the human non-pancreatic secretory PLA_2_, that is, the enzyme isoform likely responsible for PL prodrug activation in the human body. Initial geometries of transition state complexes of PLA_2_ and PL-prodrugs were done based on X-ray structures of PLA_2_ with transition state analog (PDB code 1POC for bee venom PLA_2_ and PDB Code 1POE for human non-pancreatic PLA_2_) [45,46]. The proteins were modeled with the AMBER ff-03 force field and the TIP3P model defined water molecules. A GAFF force field with AM1-bcc partial atomic charges was set for the prodrug molecules. Vacuum energy minimization of PLA_2_ and PL-conjugates with atoms’ coordinates of lipid portion of conjugates constrained to the positions of corresponding atoms of transition state analog molecules were used to obtain the initial coordinates of the binding complexes. PLA_2_/prodrug complexes were then solvated in a periodical box of water with dimension 60 × 60 × 60 Å. To model the initial state of the prodrugs in water, the prodrug molecules were solvated in a box of water with dimension of 56 × 56 × 56 Å. To model the initial state of the prodrugs in lipid phase, an equilibrated model of bilayer of phosphatidylcholine (PC) molecules (64 × 2 PC molecules in the bilayer) was used. One of the lipid molecules was modified into the prodrug molecule. Equilibration molecular dynamics simulations were performed (1 ns, constant pressure) for the lipid and water solvated molecular systems. 

### 2.10. Thermodynamic Integration (TI) and Umbrella Sampling Calculations

TI calculations for the free energy of -CH_2_ unit removal were performed using the Hamiltonian transformation of the prodrug molecule from *N* to (*N* − 1) linker units (*V_N_* to *V*_*N* − 1_) according to Equation (3).

(3) $$V_{N}\left(\lambda \right)={\left(1-\lambda \right)}^{k}V_{N}+\left[1-{\left(1-\lambda \right)}^{k}\right]V_{N-1}$$

Coefficient *k* in Equation (3) was set to 4. The free energy integral $\Delta A=\int_{0}^{1}\langle \partial V/\partial \lambda \rangle_{\lambda }d\lambda$ was computed using 9*λ* values and Gaussian quadrature. 200 ps MD simulations were performed for each *λ* value to obtain averaged $\langle \partial V/\partial \lambda \rangle_{\lambda }$ values.

Umbrella sampling free energy calculations following the removal of one -CH_2_ unit were performed by changing the equilibrium distance for a bond that connects the drug moiety to the end of the linker. The force constants in these calculations were set to 4.0 kcal/mol/Å^2^ and bond equilibrium distances were changed from 0.5 Å to 5 Å and back from 5 Å to 0.5 Å, with an interval of 0.5 Å. MD simulations for each value of equilibrium distance were run for 2 ns to allow for protein relaxation. Statistical errors of these calculations were estimated by comparing the relative free energies of the forward and backward sweeps (hysteresis). 

## 3. Results

### 3.1. Synthesis

**Figure 3:**
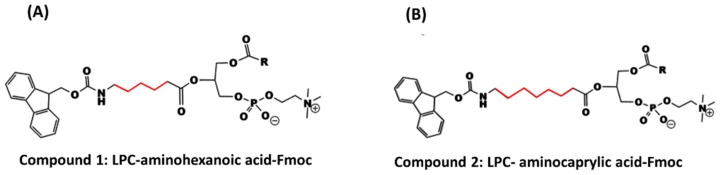
Structures of the newly synthesized LPC-Fmoc conjugates with increasing linker lengths 5 -CH_2_ units (**A**) and 7-CH_2_ units (**B**) marked in red.

In this study, two LPC-Fmoc conjugates were designed: LPC-aminohexanoic acid-Fmoc and LPC-aminocaprylic acid-Fmoc (Figure 3A,B). The synthesis was based on condensation of LPC, with linker-Fmoc involving different linkers with 5- and 7-carbon units (Scheme 1). The final product of the synthesis is the LPC-Fmoc conjugate that contains two main components, the LPC moiety covalently bound to an Fmoc group. The conjugates were purified and characterized by LC-MS and NMR. The ESI-MS spectrum showed prominent peaks at *m*/*z* 830.49; 859.52 [M−H]^−^, corresponding to protonated molecular ions (negative mode) of compounds 1 and 2, respectively. 

### 3.2. In Vitro LPC-Linker-Fmoc Conjugate Activation by PLA_2_

**Figure 4:**
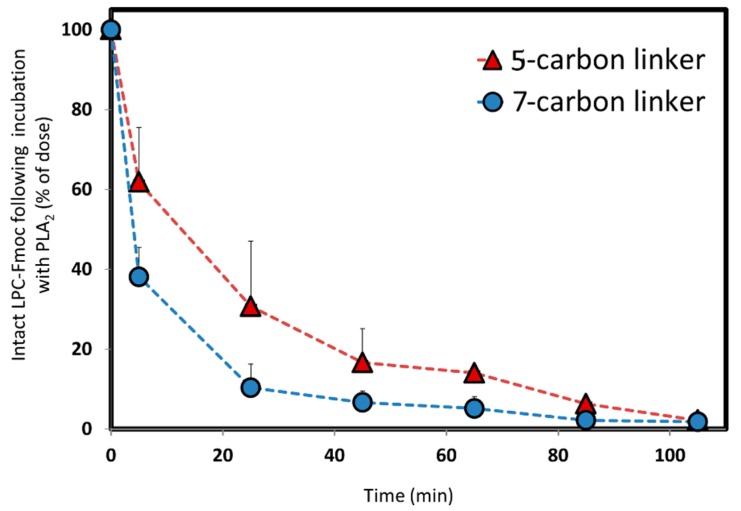
The degree of intact LPC-Fmoc conjugate (with -5 and -7 carbon unit linker) following a 105 min incubation with PLA_2_ (% of dose). Data are presented as average ± SD; *n* = 3.

The in vitro activation was presented in Figure 4, and it demonstrates the difference in the activation between the LPC-linker-Fmoc conjugates with 5- and 7-CH_2_ units when incubated with bee venom PLA_2_. The activation profile shows that the length of the linker between the LPC and the active moiety considerably influences enzyme activation, and consequently the ability to hydrolyze the ester bond between the LPC and Fmoc group. Following the period of incubation (105 min), both conjugates demonstrated complete degradation. However, it can be seen that the conjugate with 7-carbon linker has higher affinity towards the enzyme and showed greater PLA_2_-mediated activation. 

### 3.3. DSS-Induced Colitis and Colonic sPLA_2_ Overexpression

**Figure 5:**
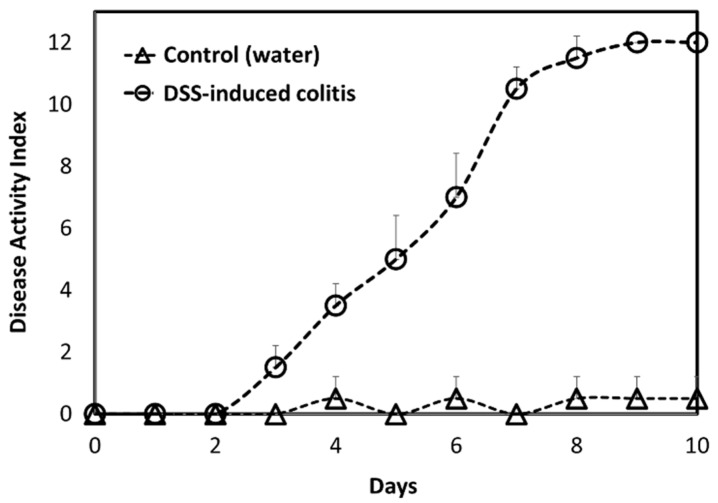
Disease activity index (DAI) score assessed daily for each animal in the dextrane sulphate sodium (DSS)-induced colitis rats (○) vs. healthy animals (∆) for 10 days. Results are expressed as mean ± SD (*n* = 2).

**Figure 6:**
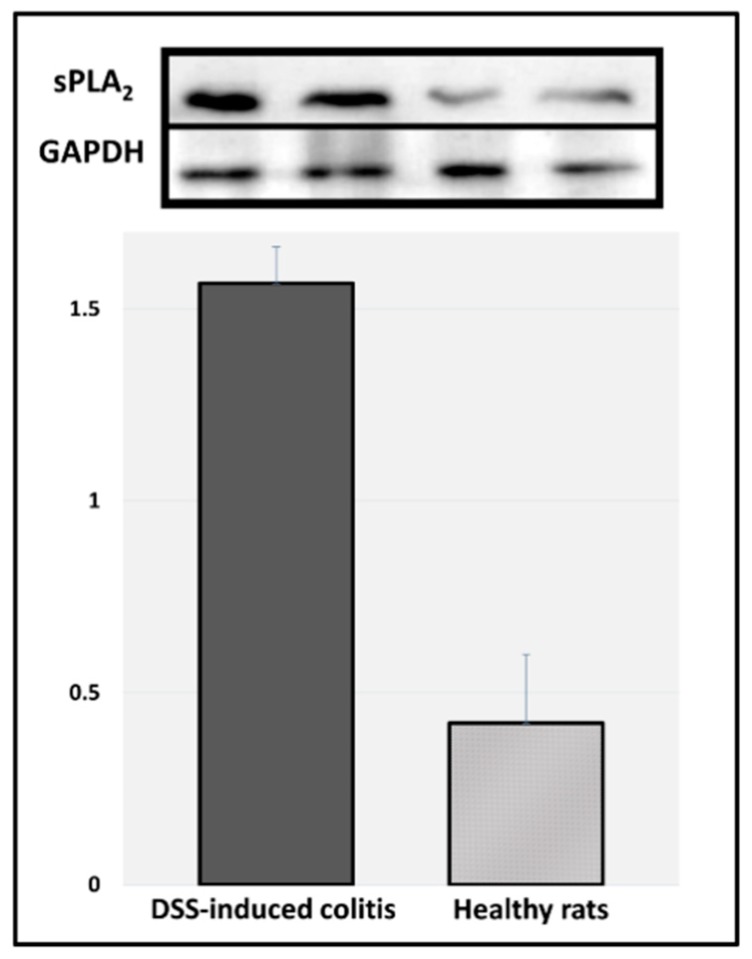
Western immunoblotting analysis of sPLA_2_ expression (vs. GAPDH) in the colonic mucosa of DSS-induce colitis rats (2 left bands) vs. healthy animals (2 right bands), and their corresponding semi-quantitative analysis computerized using Image Studio Lite Ver. 5.2.

DSS-treated and control animals were monitored daily for survival, body weight, rectal bleeding, and stool consistency. The disease activity index (DAI) was calculated as a combined score of weight loss compared to initial weight, stool consistency, and rectal bleeding. On day 10, the score resulted in weight loss >20%, diarrhea, and gross bleeding from the anus, with the score of 12 for diseased animals, vs. no symptoms and a DAI score of 0.5 ± 0.7 for control animals (Figure 5). Colon length of the diseased rats was 8.0 ± 0.1 cm, whereas the length of the colon in healthy rats was 10.1 ± 0.1 cm. The expression of sPLA_2_ was determined in the colonic mucosa of DSS-induced colitis vs. healthy rats. Immunoblotting analysis showed a ~4-fold higher expression of sPLA_2_ in the colonic tissues of diseased vs. healthy rats (Figure 6). 

### 3.4. In Silico Simulations

**Figure 7:**
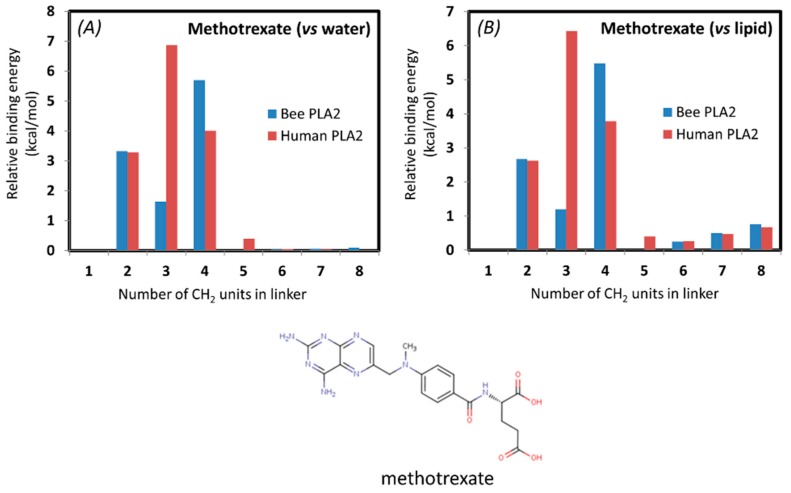
Computed values of binding free energies for the PL-methotrexate prodrugs with different linker lengths in the transition state complex with both human and bee venom PLA_2_ in water (**A**) and lipid (**B**) initial state. PLA_2_, phospholipase A_2_; PL, phospholipid. The standard error for all simulations was 0.5–1.0 kcal/mol.

**Figure 8:**
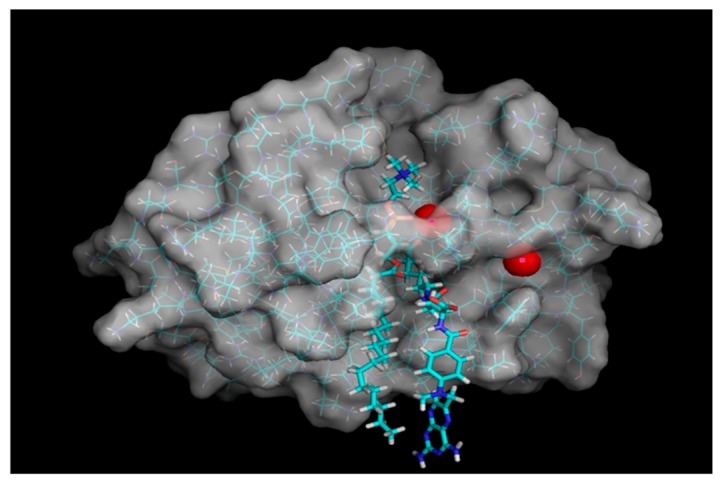
Optimized transition state geometries of human PLA_2_/PL-methotrexate prodrug complex with a 5-carbon linker. PLA_2_, phospholipase A_2_; PL, phospholipid.

**Figure 9:**
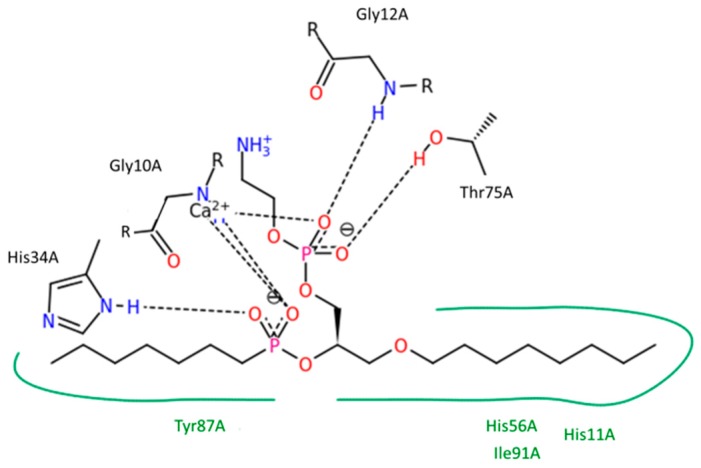
Binding pocket of bee venom PLA_2_ with a transition state analog, phospholipid [45]. PoseView diagram: Black dashed lines indicate hydrogen bonds, salt bridges, and metal interactions. Green solid lines show hydrophobic interactions and green dashed lines show π–π and π-cation interactions. Interactions are determined by geometric criteria, as previously described [47].

**Figure 10:**
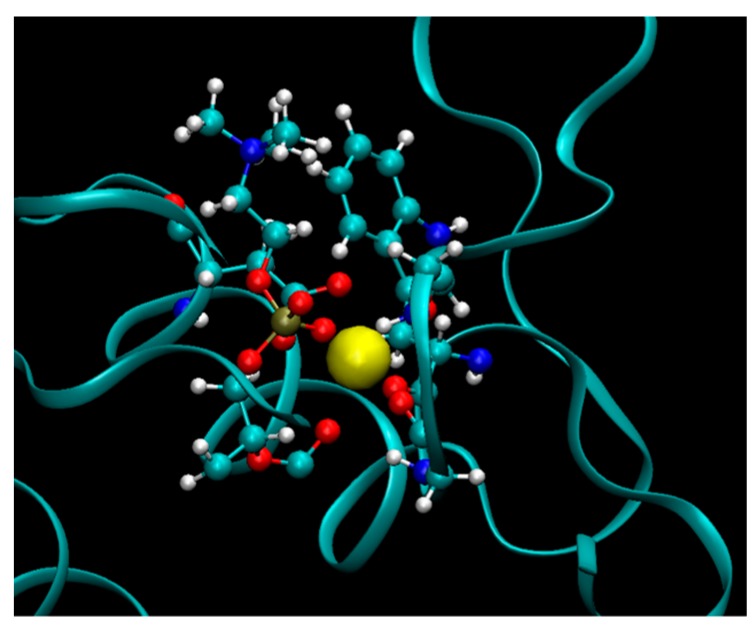
Bee venom PLA_2_ binding pocket, demonstrating the binding of the PL-methotrexate complex. For clarity, only residues within 3.0 A of the Ca^2+^ metal center are shown. The Ca^2+^ ion is ligated to tryptophan, 2 glycine, and aspartate residues, as well as the phosphate group of the PL-methotrexate conjugate. Atoms are labeled as follows carbon (cyan), hydrogen (white), oxygen (red), nitrogen (blue), phosphate (gold), and calcium (yellow). The protein backbone is shown as a cyan ribbon.

Figure 2 illustrates the thermodynamic cycle used for calculating the binding free energies of PL-methotrexate conjugates with differing linker lengths in the transition state of PLA_2_. The results of calculations of $\Delta G^{tr}$ for PL-methotrexate prodrugs with linker length varying from 2 to 8 carbons on two PLA_2_ variants (bee venom PLA_2_ and the human non-pancreatic secretory PLA_2_) are presented in Figure 7A,B for the water and lipid phases, respectively. Table 1 and Figure 7 clearly show that PL-methotrexate variants with short linkers (2–4 CH_2_ groups) have higher binding free energy (proportional to a lower rate of PLA_2_ hydrolysis) in the PLA_2_ transition state geometry than prodrugs with longer linkers. No significant difference was found in the calculated binding free energy for linker lengths of 5-, 6-, and 7-carbon atoms in water phase (Figure 7A), or for linker lengths of 5- and 6-carbon atoms in lipid phase (Figure 7B). Higher rates of enzyme activation are relative to lower binding free energies, therefore it is expected that the linker length of 6-CH_2_ units will show the highest extent of PLA_2_-mediated hydrolysis. Conjugates with linker lengths beyond this point demonstrated a slight increase in binding free energy (lipid state, Figure 7B). This trend was also demonstrated in previous studies [38,40]. Figure 8 demonstrates the optimized transition state geometry of human non-pancreatic PLA_2_ and a PL-methotrexate prodrug complex with a 5-carbon linker. Figure 9 shows a binding pocket of bee venom PLA_2_ with a phospholipid (transition state analog), taken from 1POC.PDB [45,46]. The binding pocket of bee venom PLA_2_ and PL-methotrexate conjugate are presented in Figure 10. 

## 4. Discussion

Various targeting approaches have been employed to deliver drugs specifically to the colon [48]. In UC, those strategies include biodegradable polymer coating, use of polymers sensitive to the difference in the colonic pH, time-dependent formulations, and biodegradable matrices, as well as forming a prodrug [49,50]. The aim of these approaches is to decrease the drug release/absorption in the upper GIT and facilitate colonic drug delivery. The role of colonic microflora is to ferment various molecules that were undigested in the small intestine. For this purpose, the microflora in the colon produces numerous enzymes like azoreductase, glucuronidase, glycosidase, dextranase, esterase, nitroreductase, and cyclodextranase, which are widely exploited in colon-targeted drug delivery [51]. Besides the azo-bond, colon-targeting prodrugs can contain a number of different chemical bonds, such as amide, carbamate, glycosidic, and glucuronide linkage [49]. The enzymes trigger a drug’s release from the drug carrier, or in the case of prodrugs, the enzymes can trigger the release of the free drug moiety from the prodrug molecule. 

The inflammation in UC patients is mainly located in the colon [52]. Previous reports show that there is an overexpression of the enzyme PLA_2_ in the inflamed tissues of UC patients [21,22,23,24,53]. In this study, we aim to employ PLA_2_ as a prodrug-activating enzyme, and we hypothesize that it would liberate the free drug moiety specifically to the targeted, inflamed colonic tissues (Figure 1). PLA_2_ is responsible for hydrolysis of the fatty acid in the *sn*-2 position of the PL, thereby liberating the free fatty acid and an LPC. It has been reported that PLA_2_ requires a fatty acid in the *sn*-2 position in order to achieve activation. Nevertheless, in our previous reports, we show that even though this is correct for direct conjugation between the PL and the drug [54], if a linker is introduced between them, depending upon the linker length, different extents of PL-drug hydrolysis are possible [32,39,41]. For instance, for PL-diclofenac prodrugs, it was shown that the linker length of 6-carbon atoms provides the highest activation rate, whereas lower linker lengths decrease the affinity towards PLA_2_, and even higher linker lengths failed to improve the activation further, but rather decreased it [32,40]. In addition, it was previously demonstrated that the intact PL-prodrug of indomethacin does not get absorbed into the systemic circulation, but rather remains within the intestinal lumen to be activated by PLA_2_ enzyme, hence, releasing the free drug that can then be absorbed [39]. Hence, the PL-prodrug complex can travel through the entire GIT, until it reaches the sites of inflammation (PLA_2_ overexpression), where it will be activated. 

A DSS-induced animal model of acute colitis represents a good animal model for colonic inflammation [42]. Inflammation was confirmed with a DAI score for diseased vs. healthy animals (Figure 5). Indeed, it was observed that the intestinal inflammation in this model was visualized mostly in the colonic tissue. Immunoblotting results of sPLA_2_ expression in the colonic tissues of DDS-induced colitis vs. healthy rats point to the fact that these inflamed tissues have significantly higher expression of this enzyme (Figure 6). These results validate the hypothesis proposed in this study: a careful design of a PL-drug conjugate that can be activated by PLA_2_ will result in the liberation of the parent drug from the prodrug complex specifically at the diseased colonic tissue, where PLA_2_ is overexpressed. This novel colitis-targeting prodrug approach provides exciting opportunities in the modern treatment of inflammatory bowel disease. 

We designed and synthesized a conjugate of PL and Fmoc, with different linker lengths of 5- and 7-carbons (Scheme 1, Figure 3). Fmoc is a model compound, used for validating our hypothesis: the PL-drug conjugate can be activated in the presence of high levels of PLA_2_, and depending upon the length of the linker between the PL and the drug moiety, different rates of activation are achieved. The in vitro activation presented in Figure 4 shows that change in only 2-CH_2_ (between 5- and 7-carbon linkers) demonstrate different release profiles when the PL-Fmoc conjugates were incubated with PLA_2_. PL-Fmoc conjugate with 7-carbon linker demonstrated a higher activation profile than the conjugate with the 5-carbon linker. These results support our hypothesis that once the drug moiety is conjugated to the PL, we could design an optimal linker between the drug and the PL in order to obtain the highest degree of PLA_2_-mediated activation. For drugs with severe side effects (such as methotrexate), it could highly influence the dose administered and reduce the drugs’ side effects.

In addition, an In-Silico study was conducted for methotrexate, a drug already in use for UC, in order to determine the optimal linker for enzyme degradation. The simulation of the PL-methotrexate conjugates with differing linker lengths was conducted in order to confirm previously presented in vitro proof-of-concept. For the first time, in silico calculations were conducted on a drug already in use for UC. From the calculations based on thermodynamic cycles presented in Figure 6, it is evident that the steric interactions (clashes) between the drug moiety and the PLA_2_ are highly increased for prodrug molecules with short linkers. The steric interaction of the drug moiety with the enzyme appears in the calculations as an increased free energy, pulling the drug molecule to the end of the linker in the protein. Simulations were done with both bee venom and human PLA_2_ in order to show the biorelevance of using the bee venom enzyme in in vitro studies as a substitute for human PLA_2_. The correlation between the two enzymes was demonstrated previously [38,40]. The interactions of methotrexate with human and bee venom PLA_2_ seem to differ substantially in comparison to previously studied PL-prodrugs [38,40], where a higher degree of correlation was found between the binding free energy of the PL-prodrug to the two different enzymes (Table 1, Figure 7). This can be due to the strong electrostatic interactions of the negatively-charged methotrexate molecule with the calcium ion in the active site of PLA_2_. Methotrexate prodrug derivatives exhibit drops in the transition state free energy for very short linkers (2 units), due to the stabilization of electrostatic interactions. PL-methotrexate conjugates are hydrophobic, and we can reasonably assume that the prodrugs reside in a free initial state of lipid phase, rather than water. Changing the reference state from water to lipid for PL-methotrexate conjugates (Table 1 and Figure 7) changes the relative PLA_2_ transition state transfer free energies for prodrug molecules with longer linkers (6–8 units). The conjugates with longer linkers are more difficult to transfer from lipid to PLA_2_ due to hydrophobic interactions. This appears in calculations as lowered free energy to destroy the CH_2_ group of the PLA_2_/prodrug complex, rather than as a prodrug molecule in the lipid bilayer. It can be concluded that for PL-methotrexate conjugates with shorter linkers, there is a decrease in activation due to the steric hindrance, whereas the linker length of 6 is considered the optimal one (Figure 8). It can be hypothesized that linkers longer than the optimal one has no further impact on the activation rate of the enzyme. Or, that on the contrary, longer linker lengths exhibit a slight increase in binding free energy when the conjugate is in the lipid initial phase, which can be explained by hydrophobic interactions that hinder the transfer from the lipid phase to the PLA_2_. A similar trend was demonstrated previously [38,40], and experimentally confirmed [32]. Altogether, it is clear that a linker length between the PL and the drug moieties is vital for optimal prodrug activation, and both shorter and longer linkers result in suboptimal drug release. This study further confirms the trend of different PLA_2_-mediated rates of activation for conjugates with different linker lengths, as demonstrated for the newly synthesized model compound PL-Fmoc.

We hypothesize that due to PLA_2_ overexpression in the inflamed colonic tissue of UC patients, significant peaks of the free drug would be obtained in the inflamed tissues exclusively. This may lead to elevated drug levels in the diseased tissue(s) only, not affecting the rest of the healthy intestine. Additionally, the fact that this conjugate complex does not penetrate the intestine and has negligible bioavailability may lead to efficient release of the drug in the colonic inflamed tissues. This approach provides a novel colonic drug-targeting approach in UC therapy. In addition to this, such approach can also be employed in other conditions with PLA_2_ overexpression, such as colon cancer [29].

## 5. Conclusions

This work demonstrates that for carefully designed PL-drug conjugates, PLA_2_ overexpression in UC inflamed colonic tissues can be used as prodrug-activating enzyme and drug-targeting strategy. Significant insights into the prodrug activation mechanism in the PLA_2_ binding site were revealed. This approach has the potential to provide optimized drug therapy and improved overall patient care in IBD.

## Figures and Tables

**Table 1:** Binding free energies of PL-methotrexate prodrugs in bee venom vs. human PLA_2_ transition state from water or lipid (kcal/mol), as calculated by the thermodynamic cycle illustrated in Figure 2. All simulations had standard error of the mean (SE) in the range of 0.5–1.0 kcal/mol.

Linker Lenghts	Lipid (kcal/mol)		Water (kcal/mol)	
	Bee Venom PLA_2_	Human PLA_2_	Bee Venom PLA_2_	Human PLA_2_
C2	2.7	2.6	3.3	3.3
C3	1.2	6.4	1.6	6.9
C4	5.5	3.8	5.7	4.0
C5	0.0	0.4	0.0	0.4
C6	0.3	0.3	0.0	0.1
C7	0.5	0.5	0.1	0.0
C8	0.8	0.7	0.1	0.0
